# Pediatric linear cutaneous lupus mimicking lichen striatus

**DOI:** 10.1016/j.jdcr.2026.06.051

**Published:** 2026-06-27

**Authors:** Jiaming Wang, Ning Xu, Zizhuo Li, Bo Yu

**Affiliations:** a Department of Dermatology, Peking University Shenzhen Hospital, Shenzhen, China; b Shantou University Medical College, Shantou, Guangdong Province, China

**Keywords:** dermoscopy, histopathologic examination, lichen striatus, linear cutaneous lupus erythematosus

## Introduction

Linear cutaneous lupus erythematosus (LCLE) is an extremely rare subtype of cutaneous lupus erythematosus (CLE), with the majority of reported cases occurring on the face.^1^ Its skin lesions typically lack classic discoid features and follow the lines of Blaschko, a distribution pattern that generally suggests somatic mosaicism.^2^ We report a rare case of an 8-year-old patient presenting with linear flat papules on a unilateral lower extremity along Blaschko's lines, which initially mimicked lichen striatus (LS) and progressed following high-altitude ultraviolet (UV) exposure.

## Case report

An 8-year-old Chinese boy presented with a 5-year history of asymptomatic, skin-colored to pale brown flat papules distributed along the lines of Blaschko on the medial aspect of his right lower leg. Three years prior, dermoscopy suggested a possible diagnosis of LS. However, topical pimecrolimus and desonide yielded no significant improvement. Since then, it had been stable for a long time without new skin lesions. Over a year ago, following a trip to Tibet, a high-altitude place with strong UV radiation, the lesions gradually increased and extended to the right groin. He had a history of eczema, but no systemic symptoms or relevant family history.

Initial clinical presentation consisted of skin-colored, pinkish, or pale brown flat-topped papules, tightly arranged in an intermittent linear configuration along Blaschko’s lines on the medial aspect of the right lower leg (Fig 1, *A*). Dermoscopy showed red-white streaks on a pale red background with radially distributed punctate and glomerular vessels (Fig 1, *B*).

**Fig 1:**
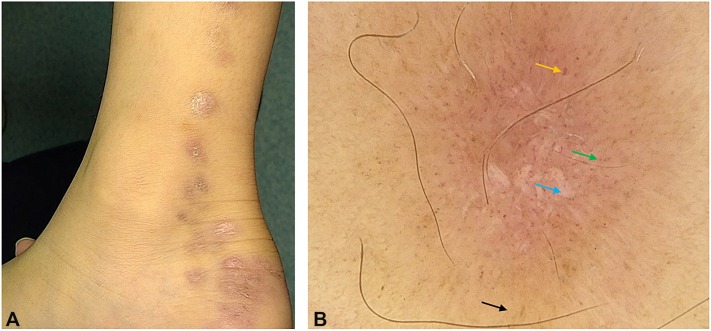
Clinical and dermoscopic images from 3 years prior. **A,** Clinical image of the lower leg at age 5 years showing skin-colored, pinkish, or pale brown flat-topped papules arranged in an intermittent linear configuration along Blaschko’s lines on the medial aspect of the right lower leg. The lesions exhibit a mildly lustrous surface. **B,** Dermoscopy of lesions reveals red-white streaks (*blue arrow*) on a pale erythematous background, accompanied by radially distributed *dotted* (*green arrow*) and glomerular (*yellow arrow*) vessels, with peripheral brown pigmentation (*black arrow*).

Current physical examination revealed dark red and brown linear atrophic plaques with mild hyperkeratosis and central atrophy (Fig 2, *A*). Subsequent dermoscopy of older lesions demonstrated white structureless areas, skin atrophy, and the “rosette sign” (4 white points in a cloverleaf-like pattern). Histopathologically, this sign corresponds to follicular plugging and infundibular hyperkeratosis (Fig 2, *B*).^3^ In the context of linear inflammatory lesions, this presence serves as a robust diagnostic clue favoring discoid-type lupus erythematosus over other blaschkoid dermatoses. Newer lesions exhibited central erythema, follicular plugging, and perifollicular white halos (Fig 2, *C*). Histopathologic examination revealed an atrophic epidermis, interface dermatitis with basal layer liquefaction, and perivascular lymphocytic infiltration in the superficial and deep dermis (Fig 2, *D*). Prominent thickening of the basement membrane zone is delineated by PAS staining (Fig 2, *E*), complemented by Alcian blue-positive mucin deposits within the deep dermis (Fig 2, *F*). Furthermore, immunohistochemistry for CD123 identifies a population of plasmacytoid dendritic cells interspersed among lymphocytes (Fig 2, *G*). Laboratory tests, including CBC, urinalysis, CRP, ESR, complement levels, anti-dsDNA, ANA, and ENA, were unremarkable, ruling out systemic involvement. In view of the patient's age and the relative stability of the cutaneous lesions, therapy with topical tacrolimus ointment was initiated. At the 2-month follow-up, the patient showed partial clinical response, with significant softening of some plaques while others remained palpably infiltrated. The patient remains under active dermatologic surveillance.

**Fig 2:**
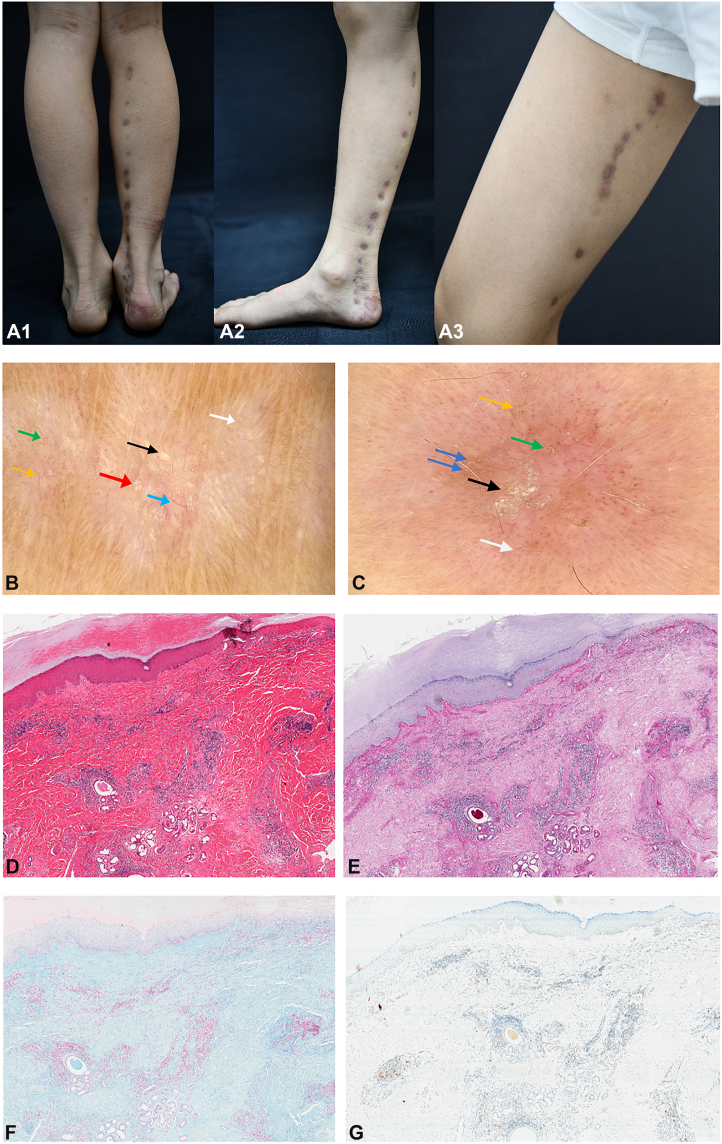
Clinical, dermoscopic, and histopathologic images at current presentation. **A1-A3,** Clinical images of the lower leg from the posterior view at age 8 years, demonstrating dark red and brown linear atrophic plaques with mild hyperkeratosis and central atrophy, featuring well-defined borders along Blaschko’s lines on the medial right lower extremity. **B,** Dermoscopy of chronic lesions demonstrates skin atrophy and whitening (*white arrow*), white structureless areas (*black arrow*), and an irregular distribution of fine curved linear (*green arrow*), arborizing (*yellow arrow*), and atypical dilated (*blue arrow*) vessels. Follicular ostia are absent, and the “rosette sign” (4 white points in a cloverleaf-like pattern, *red arrow*) is observed. **C,** Dermoscopy of new lesions shows central erythema with sparse yellow-white scales (*black arrow*), follicular plugging (*green arrow*), perifollicular white halos (*blue arrow*), multicyclic erythema (*white arrow*), and irregular pigmentation (*yellow arrow*). **D,** Histopathology of the right ankle reveals hyperkeratosis and an atrophic epidermis. Interface dermatitis, liquefactive degeneration of the basal cell layer, a few melanophagocytes in the superficial dermis, and perivascular and periadnexal lymphocytic inflammatory infiltrate in the superficial and deep dermis are present (original magnification ×40). **E,** PAS staining highlights prominent basement membrane thickening (original magnification ×40). **F,** Mucin staining reveals interstitial mucin deposition within the dermis (original magnification ×40). **G,** Immunohistochemical analysis for CD123 demonstrates plasmacytoid dendritic cells (PDCs) aggregating in clusters within perivascular and periadnexal regions (original magnification ×40).

## Discussion

Blaschko’s lines reflect the migration paths of epidermal cells during embryonic development.^2^ Inflammatory dermatoses following these lines, such as LCLE and LS, show significant morphological overlap in children. Early LCLE often presents as smooth erythematous papules without the classic disk-like elevation or follicular plugging of discoid lupus erythematosus. In dermoscopy, typical follicular plugs and white halos seen in adults may be absent in pediatric cases,^4^ though a “red starburst” pattern has been reported.^5^ Although both LS and LCLE follow Blaschko’s lines, LS typically presents histologically with perieccrine lymphocytic infiltration, lacks significant liquefactive degeneration of the basal layer or basement membrane thickening. In our case, perivascular and periadnexal inflammation extending to the deep dermis, definitively established the diagnosis of LCLE.^6^

In high-altitude environments characterized by intense UV exposure,^7^ these cell populations with genetic susceptibility along the lines of Blaschko are more susceptible to an immune-inflammatory cascade, leading to the phenotypical manifestation of a previously occult mosaic state.^8^^,^^9^ While LCLE was previously thought to lack significant photosensitivity, cases triggered by sunlight have been documented.^1^^,^^6^ Reagin et al hypothesized that the low prevalence of photosensitivity observed in blaschkoid lupus may stem from the predominance of patients of Asian descent in existing case series, as prior studies identified a lower frequency of photosensitivity among Japanese cohorts with classic cutaneous lupus.^10^ Notwithstanding the Asian ethnicity of the present patient, the documented history of intense UV exposure preceding clinical progression underscores the necessity of re-evaluating the correlation between LCLE and photosensitivity. In children, Blaschko-linear lesions are often presumed to be LS, but photosensitivity and disease persistence are critical clues for LE. This case highlights the necessity of continuous monitoring and histopathologic correlation to manage atypical pediatric linear dermatoses.

## Conflicts of interest

None disclosed.
